# A‐waves increase the risk of developing neuropathy

**DOI:** 10.1002/brb3.760

**Published:** 2017-07-12

**Authors:** Iva Srotova, Eva Vlckova, Ladislav Dusek, Josef Bednarik

**Affiliations:** ^1^ Department of Neurology Masaryk University and University Hospital Brno Brno Czech Republic; ^2^ Institute of Biostatistics and Analyses Masaryk University Brno Czech Republic

**Keywords:** A‐wave, electromyography, F‐wave, late response, nerve conduction study, neurography, neuropathy

## Abstract

**Introduction:**

A‐waves, which are observed following the M‐wave during motor nerve conduction studies (NCS), are late responses that are frequently found in many types of neurogenic disorders. However, A‐waves are also common in healthy individuals, where their significance remains unclear. The aim of this study was to examine whether the occurrence of A‐waves does in fact represent an increased risk for the future development of changes upon NCS or needle electromyography (EMG) in the corresponding nerve.

**Methods:**

Nerve conduction studies/needle electromyography findings at control examination were evaluated in relation to the occurrence of initial A‐waves in 327 individuals who had undergone repeated NCS/EMG examination and exhibited normal initial findings, with or without the occurrence of A‐waves as the only acceptable abnormality.

**Results:**

The odds ratio, which reflects the predictive power of the occurrence of A‐waves at the initial testing for the development of an abnormality (neuropathy or radiculopathy) at the follow‐up examination, ranged from 2.7 (*p* = .041) in the tibial nerve and 3.9 (*p* = .034) in peroneal one, to 30.0 (*p* = .002) in the ulnar nerve.

**Conclusions:**

A‐waves constitute an initial abnormality in all nerves, and they may be predictive for the future development of broader NCS/EMG abnormalities in the corresponding nerve.

## 1 INTRODUCTION

**Figure 1:**
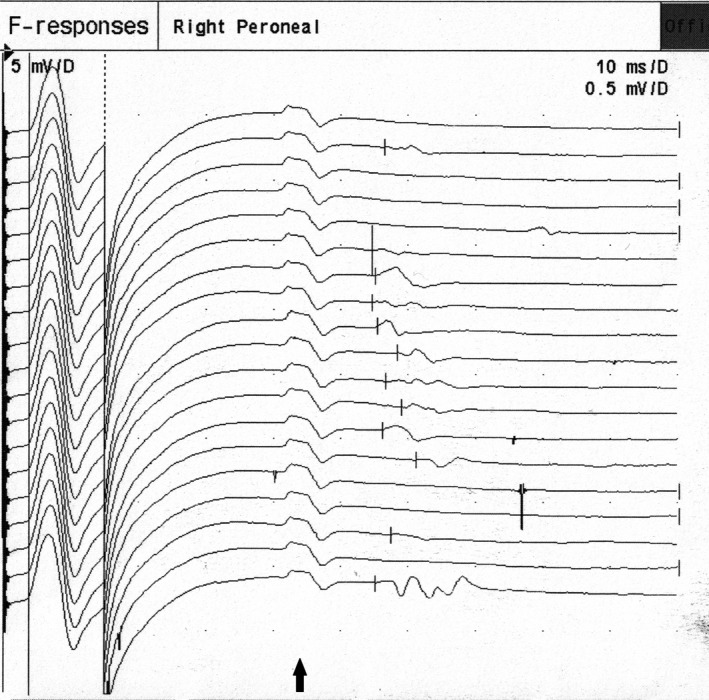
A‐waves (marked by black arrow) occurring between M‐wave and F‐wave in peroneal nerve in a healthy volunteer. Constant shape with minimal variability of the onset latency can be seen

A‐wave is a common late response, i.e., a response that can be identified following the compound muscle action potential (CMAP, M‐wave) during routine motor nerve conduction studies (NCS) (Bischoff, 2002). In general, several types of late responses can be differentiated based on their latency, amplitude, stability, the stimulus intensity necessary to elicit the response, the consistency of the occurrence and the possibility to block them using double stimulation (Bischoff, 2002). Some of these late responses are physiological (H‐reflex and F‐waves), whereas others are usually considered to be a sign of some underlying pathology (A‐waves). A‐waves are characterized by a constant shape and latency upon repeated stimulation (the variation of their onset latency is usually less than 1.5 ms) and, in most cases, a low amplitude (Figure 1). The number of A‐waves increases with an increasing stimulus intensity, whereas the latency does not change. They are frequently found in many neurogenic disorders, including demyelinating neuropathies, axonal neuropathies, focal mononeuropathies, radiculopathies, and motor neuron diseases (Andersen, Stålberg, & Falck, 1997; Bischoff, Stålberg, Falck, & Puksa, 1996; Kornhuber, Bischoff, Mentrup, & Conrad, 1999; Puksa, Stålberg, & Falck, 2003; Rowin & Meriggioli, 2000) but they are also common in healthy individuals, mainly in the lower extremities (Bischoff et al., 1996; Puksa et al., 2003; Rowin & Meriggioli, 2000). In the study published by Puksa et al. (2003), A‐waves were found in 25% of the tibial nerves studied and in 14% of the peroneal nerves in a group of otherwise healthy subjects (Puksa et al., 2003). The frequency of A‐waves in their study increased with age. Therefore, the authors hypothesize that A‐waves may be related to normal age‐related mild neuropathic changes of alpha motor neurons (Puksa et al., 2003). A‐waves thus seem to represent an initial peripheral nerve abnormality. It is, however unclear, if their presence increases the risk of development of more pronounced neuropathy in future, which—according to our knowledge—has not been studied yet.

The aim of this study was to examine whether the occurrence of supramaximally stimulated A‐waves, which were recorded as the only abnormality in routine F‐wave studies, might predict an increased probability for the development of other electrophysiological abnormalities in the respective nerve.

## 2 METHODS

### 2.1 Patients

The occurrence of A‐waves was studied retrospectively in the records of EMG/NSC examinations from patients/healthy controls performed in the electrophysiological laboratory of University Hospital Brno (which represents the biggest neuromuscular center in the Czech Republic). The use of these data for this study was approved by the Institutional Ethics Committee. Initially, one examinator (IŠ) went through all the records from the period between 2006 and 2015, and selected the patients/controls, complying with following inclusion criteria: (i) normal initial NCS and needle EMG, with or without the occurrence of A‐waves as the only acceptable abnormality, and (ii) re‐examination of the same peripheral nerve within 12–60 months. In the next step, clinical records of all the selected patients/controls were checked and individuals suitable for further analysis were selected based on the following exclusion criteria: (i) known risk factors for peripheral neuropathy (e.g., diabetes mellitus, chronic alcohol abuse, uremia, and thyroid dysfunction) and (ii) acute sensory symptoms (with the duration of few days, possibly related to acute neuropathies, e.g., Guillain‐Barre syndrome—only two patients otherwise complying with the inclusion criteria, were excluded for this reason). The majority of the individuals (102) were healthy volunteers who were examined for the purposes of several other studies. In addition, patients with no objective signs of peripheral neuropathy but with various other types of complaints (mainly subacute or chronic sensory symptoms in the upper or lower extremities [91], unexplained fatigue [47] and/or pain radiating from the cervical/lumbar spinal cord to the upper/lower extremities [87]) who had undergone re‐evaluation for the same or different reasons were included in the analysis. All these individuals (as well as the healthy volunteers) showed normal clinical neurological examination (including the evaluation of muscle strength using the MRC scale, the examination of deep tendon reflexes and sensitivity to touch, pinprick, vibration, and statesthesia and kinesthesia). The only acceptable deviation from the fully normal clinical status was the decrease (but not the absence) of Achilles tendon reflex, which was found in about one‐third of all the individuals included (Table 3 and 4). Using the above‐mentioned inclusion/exclusion criteria, a total of 815 motor nerves (median, ulnar, peroneal, and tibial) from 327 individuals (131 males and 194 females; mean age 50.3 ± 10.7 years) were further investigated for the presence of A‐waves and development of abnormities at control examination (Table 1 and 2), with a mean interval between examinations of 26.6 ± 14.2 months.

**Table 1:** Occurrence of A‐waves and its influence on the future development of NCS/EMG abnormalities in the median and ulnar nerves

Characteristics^a^	Median nerves (*N* = 188)	Ulnar nerves (*N* = 190)
Interval between 1st and 2nd examination (months)	27.5 (12.0; 60.0)	27.0 (12.0; 60.0)
Nerves with A‐waves (initial examination)	4 (2.1%)	4 (2.1%)

	Nerves with initial A‐waves (*N* = 4)	Nerves without initial A‐waves (*N* = 184)	*p* b	Nerves with initial A‐waves (*N* = 4)	Nerves without initial A‐waves (*N* = 186)	*p* b
Abnormality at 2nd examination	2 (50.0%)	28 (15.2%)	.120	2 (50.0%)	6 (3.2%)	*.009*
Types of abnormality at 2nd examination						
ALS	0 (0.0%)	0 (0.0%)	**.014** c	0 (0.0%)	2 (1.1%)	.429^c^
PNP	1 (25.0%)	1 (0.5%)		0 (0.0%)	2 (1.1%)	
Radiculopathy C8	1 (25.0%)	1 (0.5%)		0 (0.0%)	1 (0.5%)	
CTS	0 (0.0%)	26 (14.1%)		0 (0.0%)	0 (0.0%)	
Ulnar nerve entrapment neuropathy	0 (0.0%)	0 (0.0%)		2 (50.0%)	1 (0.5%)	

a Absolute and relative frequencies for categorical variables and median and min–max range for continuous variables.

b Fisher's exact test for categorical variables; Mann–Whitney test for continuous variables and scores.

c Statistical significance for the difference in the incidence of a particular abnormality at the 2nd examination between nerves with and without A‐waves at the initial examination.

Bold values are statistically significant.

**Table 2:** Occurrence of A‐waves and its influence on the future development of NCS/EMG abnormalities in the peroneal and tibial nerves

Characteristics^a^	Peroneal nerves (*N* = 212)	Tibial nerves (*N* = 224)
Interval between 1st and 2nd examination (months)	20.0 (12.0; 60.0)	20.5 (12.0; 60.0)
Nerves with A‐waves (initial examination)	21 (9.9%)	70 (31.3%)

	Nerves with initial A‐waves (*N* = 21)	Nerves without initial A‐waves (*N* = 191)	*p* b	Nerves with initial A‐waves (*N* = 70)	Nerves without initial A‐waves (*N* = 154)	*p* b
Abnormality at 2nd examination	4 (19.0%)	11 (5.8%)	**.047**	10 (14.3%)	9 (5.8%)	*.067*
Types of abnormality at 2nd examination						
ALS	0 (0.0%)	2 (1.0%)	.999^c^	0 (0.0%)	2 (1.3%)	.353^c^
PNP	3 (14.3%)	6 (3.1%)		6 (8.6%)	6 (3.9%)	
Radiculopathy L5	1 (4.8%)	3 (1.6%)		0 (0.0%)	0 (0.0%)	
Radiculopathy S1	0 (0.0%)	0 (0.0%)		4 (5.7%)	1 (0.6%)	

ALS, amyotrophic lateral sclerosis; PNP, polyneuropathy.

a Absolute and relative frequencies for categorical variables and median and min–max range for continuous variables.

b Fisher's exact test for categorical variables; Mann–Whitney test for continuous variables and scores.

c Statistical significance for the difference in the incidence of a particular abnormality at the 2nd examination between nerves with and without A‐waves at the initial examination.

Bold values are statistically significant.

### 2.2 Electrophysiological examination

Electrophysiological examinations were performed using a Keypoint Dantec type II or IV electromyography system (Dantec, Skovlunde, Denmark). Routine motor nerve conduction studies of the median, ulnar, peroneal, and tibial nerves, including 20 consecutive F‐waves and needle EMG from related muscles, were performed using standard protocols, and were processed relative to our own reference values, which are based on age and height (Kadanka, Bednarik, & Vohanka, 1994) and are similar to the reference values published by Kimura (Kimura, 2013). We used standard silver/silver chloride surface electrodes (10 mm in diameter), with the active electrode placed on the belly of the muscle and the reference electrode positioned on a muscle tendon. Electrical stimulation was performed at the distal stimulation site (80 or 100 mm proximal to the active electrode in the upper or lower extremities) using repetitive stimulation at a frequency of 1 Hz, a duration of 0.2 ms (for each stimuli) and a supramaximal intensity of 10%–15% above the level needed to elicit a maximal M‐response. Patients remained fully relaxed during the examination.

Late responses were recorded in each motor nerve when stimulated at the wrist (median or ulnar) or the ankle (peroneal or tibial). The presence of A‐waves at initial examination was evaluated from the NCS records by single observed (EV) experienced in clinical neurophysiology and blinded to the findings at follow‐up and patients/controls clinical status. A‐waves were considered present if they fulfilled the following criteria: constant shape and latency (with a chronodispersion under 0.5 ms) and present in at least 40% of the recorded responses. Similar to previous studies (Bischoff et al., 1996; Puksa et al., 2003), A‐waves were considered to be a group of late responses that were different from F‐waves and the H‐reflex and were not further classified based on their pathophysiological mechanisms. In addition, compound muscle action potentials (CMAPs), motor nerve conduction velocities (NCVs), distal motor latencies (DML), minimum F‐wave latencies and needle EMG findings in related muscles were also noted at both the initial and follow‐up examination using previously described methods (Kadanka et al., 1994). These parameters and their intertrial change were analyzed statistically to establish a possible relationship with the presence of A‐waves.

### 2.3 Statistical methodology

Standard descriptive statistics were applied in the analysis, which included the median, and the min–max range for continuous variables and the absolute and relative frequencies for categorical variables. The statistical significance of differences between the groups of patients was assessed using a nonparametric Mann–Whitney *U* test and the Kruskal–Wallis test for continuous variables. Chi‐squared test and Fisher's exact test were applied to categorical variables. The predictive power of the occurrence of A‐waves at first examination with the development of an abnormality at second examination was quantified using the odds ratio (OR) and 95% confidence interval. Estimates of OR were performed using logistic regression models. The analysis was performed using SPSS 22.0.0.1 software (IBM Corporation, 2014).

## 3 RESULTS

Initially, the data from healthy controls and patients with no objective signs of peripheral neuropathy but with various other types of complaints were compared. The initial values of all the evaluated NCS parameters (amplitudes, conduction velocities, and F‐wave latencies), clinical findings as well as the occurrence of A‐waves did not shown any significant difference between these two sugroups (Table 3 and 4). For further analysis, all the individuals were thus considered as one group.

**Table 3:** Clinical status and nerve conduction studies at 1st examination and types of abnormality at 2nd examination: comparison of the healthy controls and patients with clinical symptoms in the median and ulnar nerves

Characteristics^a^	Median nerves (*N* = 188)			Ulnar nerves (*N* = 190)		
	Healthy controls (*N* = 69)	Patients with clinical symptoms^b^ (*N* = 119)	*p* c	Healthy controls (*N* = 70)	Patients with clinical symptoms^b^ (*N* = 120)	*p* c
Interval between 1st and 2nd exam. (months)	28.0 (12.0; 60.0)	25.0 (20.0; 60.0)	.11	28.5 (12.0; 60.0)	25.5 (12.0; 60.0)	.17
Clinical status at 1^st^ examination: decreased Achilles tendon reflex	21 (30.4%)	41 (34.5%)	.57	21 (30.0%)	41 (34.2%)	.55
Nerves with A‐waves (initial examination)	1 (1.4%)	3 (2.5%)	.62	1 (1.4%)	3 (2.5%)	.62
Abnormality at 2nd examination	8 (11.6%)	22 (18.6%)	.21	2 (2.9%)	6 (4.8%)	.48
CMAP at 1st examination (mV)	10.7 (6.6;17.1)	10.2 (3.9;18.7)	.16	10.4 (7.0;17.0)	11.1 (6.3;17.1)	.38
CMAP at 1st examination (Z‐score)	0.5 (−0.7;2.6)	0.4 (−2.0;3.2)	.13	−0.3 (−1.9;2.4)	0.0 (−1.9;2.5)	.38
MCV at 1st examination (m/s)	55.2 (48.0;65.6)	55.0 (48.0;69.6)	.88	56.9 (48.5;72.1)^d^	57.1 (48.4;73.9)^d^	.51
F‐M latency at 1st examination (Z‐score)	−0.8 (−3.2;1.6)	−1.0 (−3.2;1.8)	.47	0.5 (−2.6;1.8)	−0.2 (−3.3;1.7)	.08
Types of abnormality at 2nd examination						
Normal	61 (88.4%)	97 (81.5%)	.301	68 (97.1%)	114 (95%)	.712
ALS	0 (0.0%)	0 (0.0%)		1 (1.4%)	1 (0.8%)	
PNP	1 (1.5%)	1 (0.8%)		1 (1.4%)	1 (0.8%)	
Radiculopathy C8	0 (0.0%)	2 (1.7%)	.733	0 (0.0%)	1 (0.8%)	.571
CTS	7 (10.1%)	19 (16.0%)		0 (0.0%)	0 (0.0%)	
Ulnar nerve entrapment neuropathy	0 (0.0%)	0 (0.0%)		0 (0.0%)	3 (2.5%)	

ALS, amyotrophic lateral sclerosis; CMAP, compound muscle action potential; CTS, carpal tunnel syndrome; MCV, motor conduction velocity; PNP, polyneuropathy.

a Absolute and relative frequencies for categorical variables and median and min–max range for continuous variables.

b Patients with no objective signs of peripheral neuropathy but with various other types of complaints (mainly subacute or chronic sensory symptoms in the upper or lower extremities, unexplained fatigue and/or pain radiating from the cervical/lumbar spinal cord to the upper/lower extremities).

c Chi‐squared test or Fisher's exact test for categorical variables; Mann–Whitney test for continuous variables and scores.

d For the ulnar nerve, conduction velocity across the elbow was employed.

**Table 4:** Clinical status and nerve conduction studies at 1st examination and types of abnormality at 2nd examination: comparison of the healthy controls and patients with clinical symptoms in the peroneal and tibial nerves

Characteristics^a^	Peroneal nerves (*N* = 212)			Tibial nerves (*N* = 224)		
	Healthy controls (*N* = 75)	Patients with clinical symptoms^b^ (*N* = 137)	*p* c	Healthy controls (*N* = 75)	Patients with clinical symptoms^b^ (*N* = 149)	*p* c
Interval between 1st and 2nd exam. (months)	20.0 (12.0; 60.0)	20.0 (12.0; 60.0)	.76	20.0 (12.0; 60.0)	23.0 (12.0; 60.0)	.60
Clinical status at 1^st^ examination: decreased Achilles tendon reflex	23 (30.7%)	48 (35.0%)	.52	23 (30.7%)	49 (32.9%)	.74
Nerves with A‐waves (initial examination)	6 (8.0%)	15 (10.9%)	.49	21 (28.0%)	49 (32.9%)	.46
Abnormality at 2nd examination	5 (6.7%)	10 (7.3%)	.86	5 (6.7%)	14 (9.4%)	.49
CMAP at 1st examination (mV)	5.4 (2.2;15.7)	5.1 (2.1;11.6)	.45	14.3 (3.3;25.1)	13.7 (4.5;25.1)	.22
CMAP at 1st examination (Z‐score)	−0.2 (−1.6;1.9)	−0.3 (−2.0;1.9)	.50	1.0 (−1.7;3.6)	0.7 (−1.5;3.9)	.31
MCV at 1st examination (m/s)	47.9 (40.7;69.0)	46.9 (40.3;60.4)	.24	52.1 (40.7;71.2)	50.0 (40.4;70.4)	.13
F‐M latency at 1st examination (Z‐score)	−0.3 (−2.4;1.7)	−0.2 (−2.8;1.9)	.49	0.2 (−2.5;1.9)	0.3 (−2.4;2.0)	.67
Types of abnormality at 2nd examination						
Normal	70 (93.3%)	127 (92.7%)	.999	70 (93.3%)	135 (90.6%)	.615
ALS	1 (1.3%)	1 (0.7%)		1 (1.3%)	1 (0.7%)	
PNP	3 (4.0%)	6 (4.4%)	.999	3 (4.0%)	9 (6.0%)	.787
Radiculopathy L5	1 (1.3%)	3 (2.2%)		0 (0.0%)	0 (0.0%)	
Radiculopathy S1	0 (0.0%)	0 (0.0%)		1 (1.3%)	4 (2.7%)	

ALS, amyotrophic lateral sclerosis; CMAP, compound muscle action potential; CTS, carpal tunnel syndrome; MCV, motor conduction velocity; PNP, polyneuropathy.

a Absolute and relative frequencies for categorical variables and median and min–max range for continuous variables.

b Patients with no objective signs of peripheral neuropathy but with various other types of complaints (mainly subacute or chronic sensory symptoms in the upper or lower extremities, unexplained fatigue and/or pain radiating from the cervical/lumbar spinal cord to the upper/lower extremities).

c Chi‐squared test or Fisher's exact test for categorical variables; Mann–Whitney test for continuous variables and scores.

A summary of the results processed in relation to the A‐waves presence is presented in Tables 1, 2. At the initial examination, A‐waves were rare in the upper extremities, occurring in only 2.1% of all assessed ulnar and median nerves. In contrast, A‐waves were found significantly more frequently in the lower extremities, particularly in the tibial nerve (31.3%) but less frequently in the peroneal nerve (9.9%).

At the follow‐up examination 12–60 months later, the most frequent abnormality that developed in the upper extremities was mild (22 patients) to moderate (4 cases) carpal tunnel syndrome (CTS, documented by the decrease in sensory and/or motor median NCVs in the transcarpal segment of the nerve and by the increase in distal sensory and/or motor latencies), followed by ulnar nerve entrapment at the elbow (2 cases) and Guyon′s canal (1 patient) (confirmed by decrease in motor NCVs across the elbow or Guyon′s canal together with the increase of ulnar DML in the latter case, and by the decrease of the amplitudes in the ulnar CMAP and chronic axonal changes of motor unit potencials in needle EMG from ulnar muscles). Less frequently, axonal sensory motor polyneuropathy (documented by the decrease in CMAP amplitudes and/or sensory nerve action potencials as well as by the new development of chronic axonal changes of motor unit potentials in needle EMG from distal muscles), or C8 radiculopathy (with the decrease in CMAP amplitudes of the ulnar and median nerves, and related chronic needle EMG changes in appropriate muscles) were associated with the ulnar or median nerves. In the lower extremities, the most frequent abnormal findings at the follow‐up examination were polyneuropathy (with the same criteria as had been mentioned above), followed by L5 or S1 radiculopathy (confirmed by subacute or chronic axonal changes of motor unit potentials in appropriate muscles in needle EMG). In addition, one patient without initial A‐waves had amyotrophic lateral sclerosis at follow‐up (Table 1 and 2) (documented by the generalized subacute pure motor axonopathy with fasciculations in needle EMG).

In general, new abnormalities were most frequently associated with the median nerve (possibly due to the high prevalence of CTS), followed by both nerves in the lower limbs. In the peroneal and ulnar nerves, a significantly higher number of new abnormalities were observed at follow‐up in nerves with A‐waves at the initial examination. In the tibial nerve, this difference was close to significant, whereas the difference in the median nerve was not significant (Table 1 and 2).

At the group level, a comparison of the NCS parameters at the initial examination did not show any significant difference between the nerves with and without A‐waves with the only exception of tibial nerve, which showed mild but significant decrease of CMAP amplitudes in the nerves with A‐waves at the initial examination (*p* = .031) when being expressed in mV, but was insignificant when expressed as a Z‐score, i.e., a number of standard deviations below (negative)/above (positive) the mean. Furthermore, at the individual level, the Z‐score values showed that the CMAP amplitudes were within the normal range in all the nerves (data not shown). Differences in initial MCVs and F‐wave latencies between nerves with and without A‐waves were not significant (data not shown).

**Figure 2:**
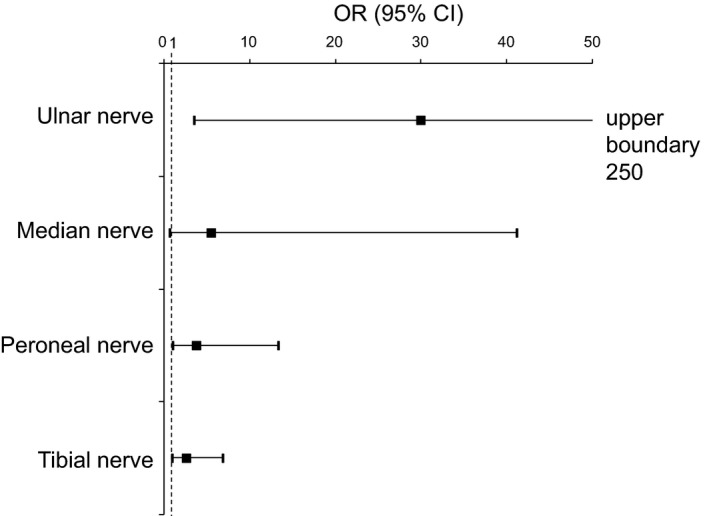
A‐wave at first examination as a predictor for abnormality at later examination. Odds ratios (OR) supplemented with their 95% confidence intervals and *p*‐values according to the logistic regression analysis showing significant predictive power of A‐waves for the development of abnormality at future examination 12 or more months later, in all the nerves with the exception of median, where such a trend achieved only borderline significance (*p *=* *.092)

The logistic regression analysis with the odds ratios ranging from 2.7 to 30.0 (*p* < .05) indicated that the presence of A‐waves at the initial examination had significant predictive power for the development of abnormalities at the follow‐up examination 12 or more months later in all nerves, with the exception of the median nerve (*p* = .092) (Figure 2, Table 5).

**Table 5:** A‐wave at first examination as a predictor for abnormality at second examination

Nerve	OR (95% CI)^a^	*p* a
Ulnar nerves	30.0 (3.6; 250.4)	**.002**
Median nerves	5.6 (0.8; 41.2)	.092
Peroneal nerves	3.9 (1.1; 13.4)	**.034**
Tibial nerves	2.7 (1.0; 6.9)	**.041**

ALS, amyotrophic lateral sclerosis; CMAP, compound muscle action potential; CTS, carpal tunnel syndrome; MCV, motor conduction velocity; NCS/EMG, nerve conduction studies/electromyography; PNP, polyneuropathy.

a Based on logistic regression.

Bold values are statistically significant.

## 4 DISCUSSION

The principal finding of this study is the confirmation that A‐waves are an initial peripheral nerve abnormality in all motor nerves, including the tibial nerve. The occurrence of A‐waves had significant predictive power for the future development of broader NCS abnormalities in part of the patients and should be considered a prognostic factor for future neuropathy, neuronopathy, or radiculopathy.

A‐waves are heterogeneous and involve several pathophysiological mechanisms, e.g., an extra‐axonal discharge elicited by an afferent action potential, hyperexcitability of proximal nerve segments, ephaptic transmission from one axon to another, or myoaxonal ephapse (Bischoff, 2002; Magistris & Roth, 1992; Roth, 1993). To distinguish the pathophysiological origin of a given A‐wave, special tests must be applied (e.g., the evaluation of the presence of A‐waves following paired or repetitive stimuli and/or during a series of stimuli applied along the course of the nerve) that are usually not conducted in routine clinical practice (Bischoff, 2002; Kimura, 2013; Magistris & Roth, 1992; Roth, 1993). During routine electrophysiological studies, underlying pathophysiological mechanisms thus are not identified, and A‐waves are considered to be a group of late responses that differ from the F‐wave and H‐reflex (Bischoff et al., 1996; Puksa et al., 2003). Therefore, to achieve a practical application of the results, the same approach was used in this study.

The patient group for this study consisted from two subtypes of individuals: healthy controls and the patients with no objective signs of peripheral neuropathy but with various other types of complaints (sensory symptoms, unexplained fatigue). There were several reasons for not including the healthy controls only. Mainly, we tried to find‐out the answer to the following question: if we perform the NCS/EMG examination in routine practice, which was completely normal with the only exception of A‐wave findings, should we pay attention to it? Does such a finding represent a subclinical ʺmild neuropathic changeʺ (as had been postulated by Puksa et al., 2003) and thus may suggest that this particular patient has an increased ʺchanceʺ to develop some clinically important abnormity in the future? This question is not related to ʺhealthy controlsʺ only, but the other way round, it may apply to any individual examined in NCS/EMG lab.

Similar to the results of previous studies in healthy individuals (Puksa et al., 2003), this analysis revealed that few A‐waves initially occurred in the otherwise normal median and ulnar nerves. However, they were more frequently observed in the peroneal nerve, and the highest number of A‐waves was detected in the tibial nerve. In this study, the frequency of A‐waves in a particular nerve at the initial examination was similar to the findings reported by Puksa et al. (2003). These findings confirm that a similar methodology for A‐wave evaluation was used in the two studies and that the individuals examined in both studies were considered to be of the ʺhealthy status.ʺ

The most frequent new abnormality found in second examination was carpal tunnel syndrome. However, to our knowledge, no study has reported a relationship between A‐waves and mild‐to‐moderate carpal tunnel syndrome; therefore, this finding may distort the results of our study because the high prevalence of CTS falsely decreases the significance and predictive power of A‐waves in the median nerve.

The significantly higher frequency of the development of new abnormalities in nerves with A‐waves at the initial examination supports the significance of A‐waves as the initial abnormality during nerve conduction studies. Based on these results, our findings show that the occurrence of A‐waves increases the future probability of developing several types of neuropathy, neuronopathy, and radiculopathy. This observation achieved statistical significance in all nerves examined, with the exception of the median nerve, for which there was only a trend towards significance, likely due to the high prevalence of CTS discussed above.

These findings were confirmed using the odds ratio and a regression analysis, which demonstrated that the presence of A‐waves at the initial examination had statistically significant predictive power for the development of abnormalities in future examinations several months later in all nerves, with the exception of the median nerve (for the same reason discussed above).

Despite statistically significant power of A‐waves in the prediction of future status of the examined peripheral nerve, the absolute numbers of the patients with newly found abnormities at second examination were quite low, which represents certain limitation of this study. Further increase in the number of patients/controls or peripheral nerves tested would allow more convincing evaluation of A‐waves significance. This, however, is not simply accessible in a single center study: the authors come from the biggest neuromuscular center in the Czech Republic with several thousands of patients examined every year. In such a big cohort, only few patients/controls complied with a strict inclusion/exclusion criteria. Further confirmation of the results by multicentre trial can provide more reliable results, which can represent a suggestion for future studies.

This study shows that the occurrence of A‐waves as a sole electrophysiological abnormality has significant predictive power for the future development of more pronounced neuropathies/neuronopathies and, therefore, provides a clear initial abnormality in the examined peripheral nerve. Our results thus suggest that the occurrence of A‐waves as a sole abnormality during the NCS/EMG examination should be reported and the re‐examination after 12 or more months might be considered in relation to clinical status of the patient. Further studies confirming the results in big cohorts of patients/controls are, however, recommended.

## CONFLICT OF INTERESTS

The authors declare no potential conflict of interests.
